# Supplementary material for: Impact of Unfortified Human Milk, Fortified Human Milk, and Preterm Formula Intake on Body Composition at Term in Very Preterm Infants: Secondary Analysis of the PREMFOOD Trial
Source: Nutrients. 2025 Apr 17;17(8):1366. doi: 10.3390/nu17081366 (PMC12030724; doi:10.3390/nu17081366)
Supplement: Supplementary file 1 [file nutrients-17-01366-s001.zip › nutrients-3377396-supplementary.pdf]

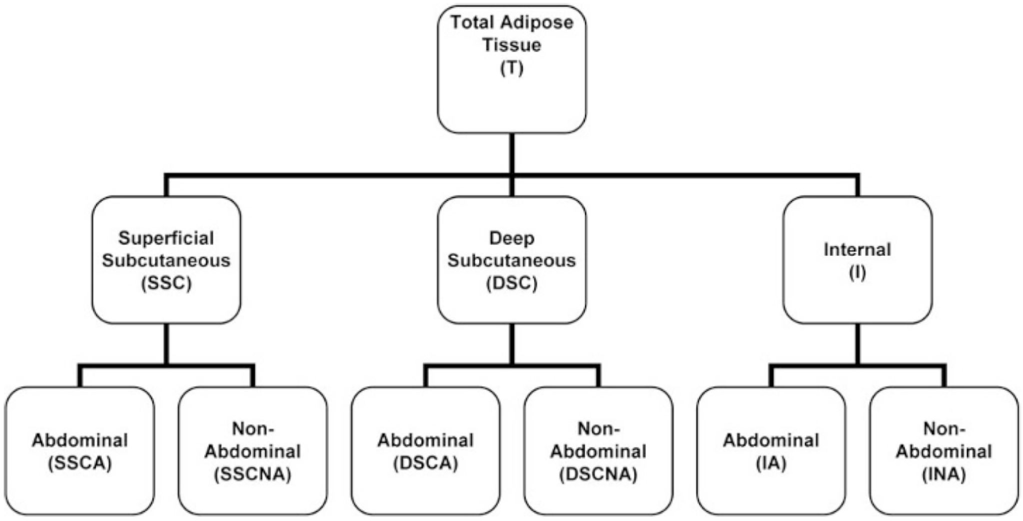

Figure S1. Classification of adipose tissue depots.

Reproduced with permission as described from: Modi N, Thomas E, Uthaya S, Umranikar S, Bell J, Yajnik C. Whole body magnetic resonance imaging of healthy newborn infants demonstrates increased central adiposity in Asian Indians. Pediatr Res. 2009;65:584–587 (ref. 37)

Table S1. Regression analyses for percentage depot adipose volume (of Total Adipose Tissue Volume) at term by feed exposure group.

		ExUHM n * = 23	PrPTF n * = 7	PrFHM n * = 17	PrUHM n * = 15
%IAAT	Unadj	Ref	-0.1 (-1.0 to 1.0), p=0.91	-0.1 (-0.8 to 0.6), p=0.84	0.1 (-0.7 to 0.8), p=0.88
	Adj	Ref	0.2 (-0.8 to 1.3), p=0.66	0.1 (-0.7 to 0.8), p=0.81	0.3 (-0.5 to 1.1), p=0.43
%INAAT	Unadj	Ref	-0.5 (-2.1 to 1.0), p=0.47	-1.1 (-2.2 to 0.1), p=0.06	-0.1 (-1.3 to 1.0), p=0.84
	Adj	Ref	-0.8 (-2.4 to 0.8), p=0.35	-1.1 (-2.2 to 0.1), p=0.07	-0.1 (-1.3 to 1.2), p=0.90
%DSCAAT	Unadj	Ref	0.2 (-0.3 to 0.7), p=0.48	0.1 (-0.3 to 0.5), p=0.70	-0.1 (-0.5 to 0.4), p=0.79
	Adj	Ref	0.1 (-0.4 to 0.6), p=0.69	0.03 (-0.3 to 0.4), p=0.86	-0.2 (-0.6 to 0.2), p=0.29
%DSCNAAT	Unadj	Ref	-0.1 (-0.6 to 0.4), p=0.66	0.1 (-0.3 to 0.5), p=0.69	0.1 (-0.3 to 0.5), p=0.74
	Adj	Ref	-0.1 (-0.7 to 0.4), p=0.62	0.1 (-0.3 to 0.5), p=0.64	0.1 (-0.3 to 0.5), p=0.64
%SSCAAT	Unadj	Ref	1.1 (-0.9 to 3.2), p=0.28	0.1 (-1.4 to 1.7), p=0.85	-0.7 (-2.3 to 0.9), p=0.37
	Adj	Ref	0.8 (-1.4 to 3.1), p=0.46	-0.05 (-1.7 to 1.6), p=0.95	-1.0 (-2.8 to 0.7), p=0.24
%SSCNAAT	Unadj	Ref	-0.7 (-2.9 to 1.6), p=0.54	0.8 (-0.9 to 2.5), p=0.34	0.8 (-1.0 to 2.5), p=0.38
	Adj	Ref	-0.4 (-2.8 to 2.1), p=0.78	0.9 (-0.9 to 2.7), p=0.32	0.9 (-1.0 to 2.8), p=0.35

* Number of infants with detailed nutritional intake available; Unadj, unadjusted; Adj, adjusted for birth gestational age, birthweight z score, sex, age at scan, and percent levels 1 and 2 care days of total stay
